# Exploratory Analysis of Methods for Automated Classification of Laboratory Test Orders into Syndromic Groups in Veterinary Medicine

**DOI:** 10.1371/journal.pone.0057334

**Published:** 2013-03-07

**Authors:** Fernanda C. Dórea, C. Anne Muckle, David Kelton, JT. McClure, Beverly J. McEwen, W. Bruce McNab, Javier Sanchez, Crawford W. Revie

**Affiliations:** 1 Department of Health Management, Atlantic Veterinary College, University of Prince Edward Island, Charlottetown, Prince Edward Island, Canada; 2 Department of Pathology and Microbiology, University of Prince Edward Island, Charlottetown, Prince Edward Island, Canada; 3 Department of Population Medicine, University of Guelph, Guelph, Ontario, Canada; 4 Animal Health Laboratory, University of Guelph, Guelph, Ontario, Canada; 5 Animal Health and Welfare Branch, Ontario Ministry of Agriculture Food and Rural Affairs, Guelph, Ontario, Canada; University of Memphis, United States of America

## Abstract

**Background:**

Recent focus on earlier detection of pathogen introduction in human and animal populations has led to the development of surveillance systems based on automated monitoring of health data. Real- or near real-time monitoring of pre-diagnostic data requires automated classification of records into syndromes–syndromic surveillance–using algorithms that incorporate medical knowledge in a reliable and efficient way, while remaining comprehensible to end users.

**Methods:**

This paper describes the application of two of machine learning (Naïve Bayes and Decision Trees) and rule-based methods to extract syndromic information from laboratory test requests submitted to a veterinary diagnostic laboratory.

**Results:**

High performance (F_1_-macro = 0.9995) was achieved through the use of a rule-based syndrome classifier, based on rule induction followed by manual modification during the construction phase, which also resulted in clear interpretability of the resulting classification process. An unmodified rule induction algorithm achieved an F_1-micro_ score of 0.979 though this fell to 0.677 when performance for individual classes was averaged in an unweighted manner (F_1-macro_), due to the fact that the algorithm failed to learn 3 of the 16 classes from the training set. Decision Trees showed equal interpretability to the rule-based approaches, but achieved an F_1-micro_ score of 0.923 (falling to 0.311 when classes are given equal weight). A Naïve Bayes classifier learned all classes and achieved high performance (F_1-micro_ = 0.994 and F_1-macro_ = .955), however the classification process is not transparent to the domain experts.

**Conclusion:**

The use of a manually customised rule set allowed for the development of a system for classification of laboratory tests into syndromic groups with very high performance, and high interpretability by the domain experts. Further research is required to develop internal validation rules in order to establish automated methods to update model rules without user input.

## Introduction

Disease emergence and bioterrorism events, especially since 2001, have highlighted some of the short-comings of traditional surveillance, generally based on laboratory test results and direct reporting [1]. Focus has shifted to earlier detection of pathogen introduction in human or animal populations, leading to the implementation of new techniques using data sources upstream to those typically used in traditional surveillance [2]; especially pre-diagnosis data that are already available and automatically collected [3], such as sales of over-the-counter medicine, absences from work or school, and patients’ chief complaint upon visits to an emergency center [4].

Due to the lack of sensitivity of pre-diagnostic data, surveillance systems using this information target general groups of diseases, or syndromes, and are therefore often referred to as “syndromic surveillance” [5]. Grouping pre-diagnostic data into syndromes is the first step of implementing a syndromic surveillance system [3]. Valid, reliable, and automatic classification of syndromes was an essential component of early computerized epidemic detection systems [6]. When data are structured using standardised codes, such as the Logical Observation Identifiers Names and Codes (LOINC®) used in laboratories, the International Classification of Diseases (now on its 10^th^ revision, ICD-10), or the Systematized Nomenclature of Medicine (SNOMED®) [7], syndrome classification can be performed by mapping those codes into syndromes. However, text mining or other machine learning tools can be invaluable when free-text or semi-structured data are being used [6]. Naïve Bayes classifiers have frequently been used in syndromic surveillance when the input data are chief complaints (free-text typed in by nurses) at emergency facilities [6], [8], [9], [10], [11].

Rule-based methods were widely used before the computational capacity of common computers made it possible for machine learning methods to be widely adopted [11]. Nevertheless, they have remained a popular choice in the health field due to their transparency and interpretability. In the 2008 challenge organized by i2b2 (Informatics for Integrating Biology to the Bedside), which consisted of automatic classification of obesity and comorbidities from discharge summaries [12], the top ten solutions were dominated by rule-based approaches, demonstrating their efficacy.

Decision trees are a third type of classification algorithm recommended when results must be delivered to a broader audience, such as health workers, as it is also an relatively simple method to interpret [13]. Other machine learning algorithms used in the medical field include: Artificial Neural Networks (ANN) [14]; and Support Vector Machines (SVM) [15]. These methods are powerful, but both adopt a “black-box” approach; so that the way in which decisions are made by the classifier is not transparent. They have been used in more complex medical tasks, such as the interpretation of radiographs and studies of drug performance [16], [17], [18]. However, to the authors’ knowledge, the use of these algorithms to classify health data for the purposes of syndromic surveillance has not been documented in the peer-reviewed literature.

In contrast to laboratory test results, on which traditional surveillance is based, laboratory test orders can be a valuable data source for syndromic surveillance, since they are collected and stored electronically in an automated manner, but are more timely for surveillance purposes than laboratory test results. Laboratory submission data have, for example, been incorporated into CDC’s BioSense Early Event Detection and Situation Awareness System [19]. Moreover, because there are fewer laboratories than sites of clinical care, the use of laboratory databases can provide more complete records and over larger areas [2]. Besides changing focus to early diagnosis, modern surveillance systems are evolving to complete *biosurveillance* systems. This term is intended to imply a broadening focus, addressing not only human health but all conditions that may threaten public health, such as a disruption in the food supply, or large social and economic disruptions resulting from outbreaks of diseases in animals [2], [20]. Besides their role in the food supply and agricultural economy, animals could serve as sentinels for the detection of certain zoonotic diseases that may be recognized earlier in animals than in humans [21].

Animal data have been incorporated into a few surveillance systems for human populations, including: the Electronic Surveillance System for the Early Notification of Community-based Epidemics (ESSENCE) [22], the North Dakota Electronic Animal health Surveillance System [23] and the Multi-Hazard Threat Database (MHTD) [24]. Glickman et al (2006) [25] and Shaffer et al (2008) [26] have investigated the value of animal health data as sentinels for public health. Despite the less frequent requests for laboratory analyses made by veterinarians compared to human clinicians, the authors hypothesized that, “the consistency of test orders over time is such that increases in cases of disease will result in detectable increases in the number of test orders submitted by veterinarians that can be identified using prospective analysis” (Shaffer, 2008 [26], page2).

An overview of the development of syndromic surveillance system in the veterinary context has been provided in a recent review of the literature [27]. This review indicated that initiatives using laboratory data had been based on establishing direct relationships between test codes and syndromic groups. The use of clinical data has typically relied on syndrome definition being provided by the veterinarian. Machine learning or rule-based methods applied to the identification of syndromes in animal health data had not been documented. This paper describes the exploratory analysis of such methods to extract syndromic information from laboratory test requests submitted to a veterinary diagnostic laboratory. These steps are part of the development of a syndromic surveillance system taking advantage of the centralized, computerized, and routinely updated sources of data provided by the Animal Health Laboratory in the province of Ontario, Canada. The initial phase of implementation, described here, focused on cattle sample submissions.

## Methods

### Data Source

The Animal Health Laboratory (AHL) at the University of Guelph is the primary laboratory of choice for veterinary practitioners submitting samples for diagnosis in food animals in the province of Ontario, Canada. The number of unique veterinary clients currently in the laboratory’s database (2008 to 2012) is 326. The AHL has a laboratory information management system (LIMS) that is primarily used for reporting the results of diagnostic tests.

Three years of historical data from the AHL were available, from January 2008 to December 2010. Cattle were chosen as the pilot species due to high volume of submissions from dairy and beef herds in Ontario. All laboratory test orders for diagnoses in cattle were extracted from the database; all farm identification elements had been removed from these data.

### Data Structure

Test requests are entered into the AHL database on a daily basis. Individual test requests are recorded as unique data entries. A common *case code* (submission number) is given to all samples from the same herd on any given day, allowing identification of samples related to the same health event. In human health, a case usually refers to one person at a time. Such that two people, with the same medical complaint, living in the same household, submitting samples on the same day would be counted as two cases. In veterinary medicine which often works in herds or flocks, samples submitted from one, two or more animals, of the same type, from the same herd (“household”) with the same medical complaint on the same day, would be counted as one case.

The nature of the diagnostic sample is identified in the database by two fields: the *sample type* field, in which the laboratory staff chose from a pre-set list (blood, feces, brain tissue, etc); and the c*lient sample ID*, a free-text field used to enter the source animal identifier given by the client. The diagnostic tests are identified by codes pre-set in the system. All codes are textual.

Table 1 shows a sample of the data. Only the fields relevant for medical information extraction are shown. Submission numbers have been removed, but samples from the same submission are represented in the table with consecutive rows in the same shading.

**Table 1:** Sample of the data available, restricted to the fields relevant for syndrome classification.

Date	Sample ID*	Client Sample ID	Sample Type	Diagnostic test code	Diagnostic test description
2010-01-04	10-####-0001	Tulip	Milk	Beta-Lactamase_Test	**Beta-lactamase_test**
2010-01-04	10-####-0002	Plum	**Milk**	Culture_Bact	Bacterial_culture
2010-01-04	10-$$$$-0005	A517_SMALL	**Intestine**	Culture_Bact	Bacterial_culture
2010-01-04	10-$$$$-0009	B516	Tissue_Pooled	RLA	**Rotavirus_A_-_latex_agglutination**
2010-01-04	10-$$$$-0010	#517,_#516	Tissue_-_Fixed	Histopathology	Histopathology
2010-01-07	10-####-0002	139_W-H-1_-_**Pericardial**	Fluid	Culture_Bact	Bacterial_culture
2010-01-07	10-####-0004	139_W-H-1_-_**Heart**	Tissue	Culture_Bact	Bacterial_culture
2010-01-05	10-$$$$-0001	Webb/None_Given	Tissue_-_Fixed	IHC_-_Bov_Corona	**IHC_-_Bovine_coronavirus**
2010-01-05	10-$$$$-0002	Webb/None_Given	Ear_-_Notch	BVDV_Antigen_ELISA	**Bovine_viral_diarrhea_virus_-_antigen_ELISA**
2010-01-05	10-####-0001	11675_BOOSTER_110004	**Semen**	Culture_Bact	Bacterial_culture
2010-01-27	10-$$$$-0031	Black_Face_w_white_spot	Blood_-_Serum	N._caninum_ELISA	**Neospora_caninum_-_ELISA**
2010-01-27	10-####-0002	**Lung**	Tissue	Culture_Bact	Bacterial_culture
2010-01-27	10-####-0003	LuLiKiSpThTy	Tissue_Pooled	Cell_Cult_Isolation	Virus_isolation_in_cell_culture
2010-01-27	10-####-0005	**Stom._content**	Tissue	Culture_Bact	Bacterial_culture
2010-01-27	10-####-0006	**liv/spl/kid**	Tissue	Culture_Bact	Bacterial_culture

* The field containing Submission ID was removed to ensure confidentiality, and omitted in the Sample ID shown.

Samples from the same case are represented in the table with consecutive rows of the same shading. Keywords and test names relevant for classification are shown in bold.

### Syndrome Definition

All of the historical data available were reviewed manually to identify the potential for syndromic classification at the time of sample submission. Veterinarians do not often provide detailed case history information. Therefore the identification of syndromes was based only on the type of diagnostic test requested, and the type of sample submitted, which allowed identification of the organ system targeted for diagnosis.

A syndromic group was defined as a group of test requests that: (i) are related to diseases from the same organ system; (ii) are all diagnostic tests for the same specific disease, in cases of tests requested so frequently that their inclusion in another group would result in their being, alone, responsible for the majority of submissions; or (iii) tests that have little clinical relevance and should be filtered out (e.g., tests in environmental samples, general haematology profiles, as well as a range of “non-specific” submissions). Despite the absence of clinical information, the sample description allows identification of abortion cases through keywords such as “placenta” or “fetus”. “Abortion” is therefore the only syndromic group defined based on a clinical syndrome, rather than using the three criteria listed above. Based on those criteria, an initial list of syndromic groups was compiled and then reviewed by a pathologist (BJM), a bacteriologist (CAM) and a clinician (DK). Following this review, all historical data were manually classified into syndromic groups to serve as training examples for the machine learning algorithms. Syndromic definition and manual classification were discussed until consensus was achieved among all experts.

Each submitted case (one or more test requests from a herd on a given day) could have multiple types of samples and/or multiple diagnostic tests requested. Syndromic classification was performed for each individual database entry (test request), and later collapsed by case submission numbers, eliminating repeated syndromes within the same case. As a result, a given case could be associated with multiple syndromes by virtue of clues relating to multiple organ systems found in the same submission.

### Mapping of Test Codes

Based on the aforementioned list of syndromic groups, a list of all diagnostic test codes that could be mapped into a syndromic group was established. Mapping is used here to describe the direct relationship: “if test requested is X, then syndromic group is Y”, and mapping rules of this type were established for all test request codes that could be classified into only one syndromic group with certainty. This is typically the case for serological tests, where the veterinarian specifies the pathogen or disease to be confirmed, and the sample type is not informative of the organ system affected, as it is “serum” or “blood”.

This mapping was built as a model in RapidMiner 5.0 (Copyright 2001–2010 by Rapid-I and contributors), an open source data mining package, which provides tools for data integration, analytical ETL (extract, transform, load), data analysis and reporting. RapidMiner includes an option to code any learned model in XML format, which can subsequently be directly manipulated.

Observations where test code was not associated with any mapping rule were assigned “Unknown” as the syndromic group at this stage in the processing. These were test requests such as “bacterial culture”, which are not informative of the disease suspicion or organ system targeted by the veterinarian. These observations formed an *unmapped* subset of the data.

### Algorithms for Automated Syndrome Classification

For the *unmapped* subset, text mining was used to separate all words found in the fields describing the sample type (*client sample ID* and *sample type*, Table 1) in the three years of available data. A tokenization process was applied using any non-letter character as a break point to separate words. The list of all mined words in the historical data was manually reviewed to construct a dictionary of medically relevant terms, as well as acronyms frequently used, and common misspellings. This is similar to the process described in [28] and [29].

Once the dictionary was built, all data tokenization was performed searching only for those specific tokens. For each observation being evaluated, the fields *sample type* and *client sample ID* were tokenized, and a vector was created to designate the binary occurrence of each word in the dictionary. These vectors were then used by the classifier algorithms to learn from the training dataset and to classify test data.

The rule induction algorithm in RapidMiner [Repeated Incremental Pruning to Produce Error Reduction (RIPPER)] was used. Information gain was used as the criterion used for selecting attributes and numerical splits. The sample ratio and pureness were set at 0.9 and the minimal prune benefit 0.25. Using the XML model of rules induced by the RIPPER algorithm as a template, a manually modified set of rules was also explored.

The Naïve Bayes learner available in RapidMiner was used to develop and apply a Naïve Bayes classifier. The learner requires no parameters settings other than an indication of whether a Laplace correction should be used to prevent high influence of zero probabilities. Laplace correction was not used.

Decisions trees were constructed using gain ratio as the criterion for selecting attributes and numerical splits. The minimal size for split was set at 4, minimal leaf size 2, minimal gain 0.1, maximal depth 20, confidence 0.25, and up to 3 pre-pruning alternatives.

The XML code of the models used, as well as the set of customised rules for classification, are available upon request from the first author.

### Assessing Algorithms Performance

Due to the large variability in the free-text entered by veterinarians to describe the samples submitted, it was deemed important to have a large test set, in order to assure that classification would be satisfactory once applied to new data. Manually classified historical data were split in half. After sorting sample submissions according to date and submission number, observations were alternately assigned to two different sets. Each classification algorithm was trained using one of the two sets, and then used to classify the alternative set. The process was then repeated switching training and test subsets.

Based on a comparison to the manual classification which had been carried out with the help of experts, the following performance measures were assessed for each classifier (using overall results from both test datasets): recall (the fraction of relevant instances correctly identified by the algorithm); precision (the fraction of the identified instances that were correct), and F_1_-score, the harmonic mean of recall and precision; i.e. (2 * precision * recall) * (precision+recall)^−1^. After computing recall, precision and F_1_-score for each of the classes, these measures were averaged over all classes to give macro-averaged scores. An average weighted according to the number of records in each of the classes was also calculated; often referred to as micro-averaged scoring.

Stability was investigated by producing slightly different training subsets (for instance removing small random samples from the training set, or eliminating individual syndromic groups at a time), and assessing the resulting difference in the performance of the classifier.

## Results

The three years of historical data contained 23,221 cases (samples from the same herd on a given day), consisting of a total of 218,795 individual test requests from cattle (i.e. bovine, dairy or beef animals of any age).

Based on an evaluation of these three years of historical data, and input from experts, the syndromic groups listed in Table 2 were defined. The table also lists the criteria for syndromic group creation and the number of test requests and cases assigned to each syndromic group following manual classification.

**Table 2:** Syndromic groups, defined based on an evaluation of three years of diagnostic test requests.

Syndromic group	Criteria for syndromic group creation	Number of test requests	Number of cases
Abortion	Clinical sign	559	225
Circulatory	Organ systems	57	50
Eyes and ears		37	20
GIT		8,733	2,564
Haematopoietic		231	199
Hepatic		135	119
Mastitis		49,246	6,766
Musculoskeletal		233	149
Nervous		150	129
Reproductive		857	192
Respiratory		8,501	1,452
Skin and Tegument		14	7
Systemic		3,328	700
Urinary		501	146
BSE*	Individual diseases with high number of testrequests	5,306	158
BLV		34,468	3,321
BVD		12,689	2,354
Johnes disease		11,123	2,040
Neosporosis		6,198	1,467
Clinical Pathology (hematology/biochemistry)	Other types of tests	61,059	4,282
Environmental samples		655	58
Antimicrobial susceptibility		140	33
Toxicology		6,866	955
Nonspecific samples	Samples whose syndromic group could not be determined	7,708	3,374
Total		218,795	30,760**

GIT = Gastro-intestinal tract; BSE = Bovine Spongiform Encephalopathy; BLV = Bovine Leukemia Virus; BVD = Bovine Viral Diarrhea.

* BSE test requests are large compared to counts of other test submissions that can be classified as “Nervous”.

** The number of cases after classification is higher than the initial number of cases because multiple syndromes can be identified within a single submission.

**Figure 1:**
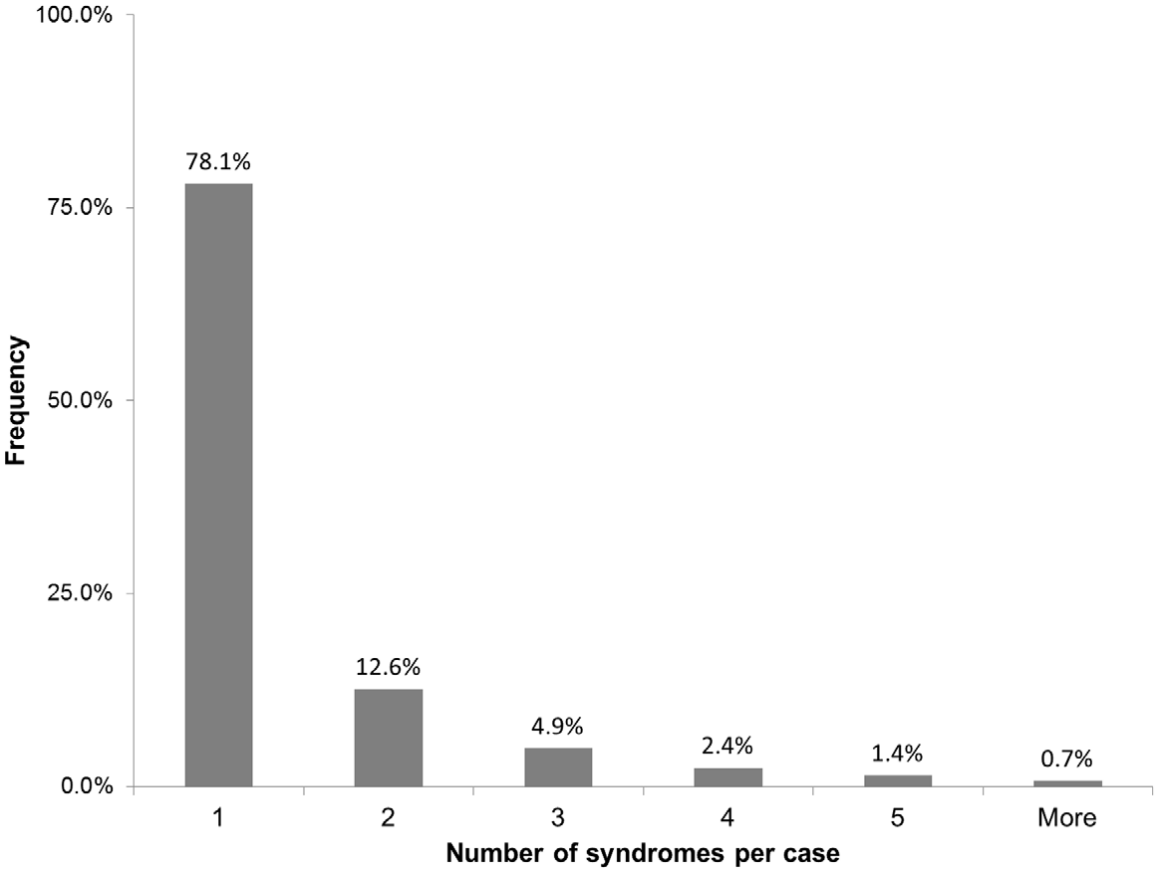
Number of syndromes identified in each case using information from individual test requests.

After classifying all sample submissions, and eliminating repeated syndromic instances within the same case, the final number of “syndromic cases” in the historical dataset was 30,760. Given that there were 23,221 initial herd investigations, this implies an average of 1.32 recorded syndromes per case. The distribution of syndromes per case is shown in Figure 1.

Of all the samples submitted, 75.7% (165,649) could be directly mapped into syndromic groups based on the test request information alone.

**Figure 2:**
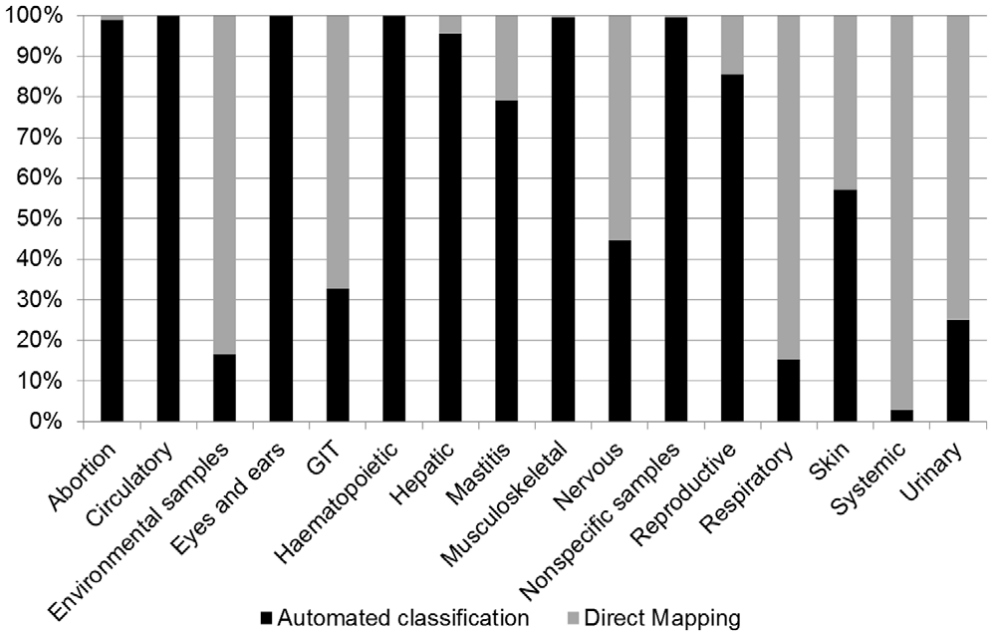
Percentage of test requests classified by direct mapping and automated classification.

For the syndromic groups created based on clinical signs, non-specific signs or specific organ systems (see Table 2), Figure 2 illustrates the percentage of test requests which could be allocated to a syndromic group via direct mapping versus those that fell into the *unmapped* subset. Around 25% (53,146) of all instances in the database could not be directly mapped into a syndromic group and these provided the material for which automated classification was explored. Although these *unmapped* instances contain 16 of the original 22 defined syndromic groups, the syndromic group “Mastitis” alone is responsible for over 70% of these instances, and three groups (“Mastitis”, “Nonspecific” and “GIT”) account for over 90% of the data, as shown in Table 3. For the groups Mastitis and GIT, 94% and 77% of the *unmapped* observations, respectively, refer to the test “Bacteria culture”. *Unmapped* observations which are ultimately classified as “Nonspecific” contain a greater variety of test names, including the following which occur frequently: “Bacterial culture” (18%), “Histology” (27%) and “Necropsy” (18%).

**Table 3:** Instances and syndromic groups in the *unmapped* subset of the data.

Syndromicgroup	Instances	Percentageof total	Cumulativepercentage
Mastitis	38,934	73.26%	73.26%
Nonspecific	7,667	14.43%	87.68%
GIT	2,857	5.38%	93.06%
Respiratory	1,309	2.46%	95.52%
Reproductive	732	1.38%	96.90%
Abortion	553	1.04%	97.94%
Musculoskeletal	232	0.44%	98.38%
Haematopoietic	231	0.43%	98.81%
Hepatic	129	0.24%	99.06%
Urinary	125	0.24%	99.29%
Envir. samples	109	0.21%	99.50%
Systemic	98	0.18%	99.68%
Nervous	67	0.13%	99.81%
Circulatory	57	0.11%	99.91%
Eyes and ears	38	0.07%	99.98%
Skin and Tegument	8	0.02%	100.00%
Total	53,146		

The results of automated classification using different algorithms are shown in Table 4 and described in detail below.

**Table 4:** Performance measures for the algorithms implemented.

	Class average (Macro)*			Weighted average (micro)		
Algorithm	recall	precision	F-score	recall	precision	F-score
Manually modified rules	.994	1.000	.997	1.000	1.000	1.000
Rule Induction**	.626	.793	.677	.991	.981	.979
Naïve Bayes	.983	.939	.955	.994	.996	.994
Decision Trees**	.290	.416	.311	.936	.937	.923

* The total number of groups in the training data was 16, and the total number of instances 53,146.

** The Rule Induction algorithms failed to learn 3 classes, and the Decision Tree 11 classes.

The use of rule induction (RIPPER) achieved only moderate performance overall. Three groups with low frequency of test requests – “Environmental samples”, “Skin”, and Eyes and Ears” – were not included in the rules, but as shown in Table 3 these groups represent only 0.3% of all instances subjected to automated classification. The F_1_-macro average was 0.677, but because the unlearned groups account for such a small proportion of the submissions, when the classes’ performance is averaged accounting for the weight of each class, the F_1-micro_ is 0.979 (Table 4). Upon manual review of the rules created by the algorithm, it was found that the main source of error was failure of the algorithm to establish good decision rules when multiple medically relevant words were found in the same test request. This method was easy to implement and the rules generated are transparent and easily interpreted.

The rules produced by the RIPPER algorithm were manually modified to account for some of the relationships missed, producing a set of custom rules. Running the custom rule set against the entire *unmapped* subset resulted in an F_1-macro_ score of 0.997, and F_1-micro_ score of 0.9995 (Table 4). The remaining errors tended to be due to use of abbreviations not common enough to have been incorporated in the rules, misspellings or the absence of a space between two words, resulting in the tokenization process failing to identify these words.

The performance of the Naïve Bayes algorithm was high (F_1-macro_ of 0.955 and F_1-micro_ 0.994), as shown in Table 4. The main performance issue associated with this algorithm was its instability. Slightly different datasets resulted in very different performances (results not shown). With unbalanced training and test datasets, for instance, rather than assigning the label “Nonspecific” to samples that could not be classified, the Naïve Bayes algorithm would assign these samples, as well as misclassified samples from other groups, into one of the groups with a small number of submissions.

The classifier based on Decision Trees performed reasonably well in the micro score (F_1-micro_ score of 0.923). However the classifier failed to learn 9 classes, which are biologically relevant, despite accounting for only 2% of the *unmapped* instances (which explains the high micro average). Moreover, the models appeared to be unstable: slight changes in the training data could result in a completely different ‘shape’ of decision tree, and a similar phenomenon was observed when the initial parameters for minimal gain and confidence where varied.

## Discussion

This study evaluated the classification of structured data from animal laboratory test requests into syndromic groups for surveillance. This type of data lacks specificity not only because it precedes diagnostic results, but also due to the limited amount of clinical information provided by veterinarians. Previous work has focused on the direct mapping of specific test requests to syndromic groups [25], [26]. Here the use of text-mining was explored to extract information from fields containing a description of the sample collected by the veterinarian, in order to identify the organ system(s) affected in the clinical case being investigated.

Due to the structured format of the data, the text-mining task did not need to account for sentence semantics or other contextual information. Statistical methods were sufficient to capture the majority of medically relevant information from the fields mined. The binary occurrence of words from a manually constructed dictionary served as input to the classifier. The algorithms needed therefore to learn the relationship between these words, their co-occurrences and the target syndromic group.

Rule induction is suitable for uncovering these types of regular relations [28], and is recommended in cases when improvements in accuracy can be achieved by incorporating relationships among attributes [30]. However, upon manual review of the rules created by the algorithm, it was found that performance could be improved by including specific relationships in cases of multiple word occurrences. It was noted that the main relationships that the rule induction had failed to capture involved:

- Sampling of multiple organs. For instance *heart* was associated with the “Circulatory” syndrome, and *liver* with “Gastro-intestinal”, but the observation of samples from both organs in the same test request should be classified as “Systemic”.
- Precedence being given to some words. “Abortion” is an actual clinical syndrome, in contrast to all other groups based on organ systems. Therefore the observation of any words related to abortion (*fetus*, *placenta*, *aborted*, etc) should result in classification of “Abortion”, regardless of what fetal organ(s) was(were) collected.
- The co-occurrence of words which have a different meaning than when they occur on their own. For instance *ear* is a word included in the dictionary of relevant terms and would typically be associated with the “Eyes and ears” syndrome; however, this word should be ignored when it appears in the expression *ear tag*, which refers only to animal identification within a herd.

These relationships are still simpler than typical contextual challenges associated with full textual analysis, and the set of manually modified rules exhibited high performance. The remaining issues that prevented correct classification, such as misspellings and inconsistent abbreviations relate to the quality of the data, something which often complicates the interpretation of syndromic information [31].

The rule-based algorithm using manually modified rules was considered the most suitable algorithm for the classification of the animal laboratory dataset at hand, due to its high accuracy, ease of implementation, and high interpretability/transparency. Although simple, this rule-based solution is in line with research reporting from the i2b2 Obesity Challenge. Among the top 10 performing systems, rule-based approaches were the most successful in the textual task, which required classification based on documented information [12].

Rules also have the advantage that they are transparent and can typically be easily interpreted by the collaborating health experts [28]. Their main disadvantage is the knowledge acquisition bottleneck, in the case where rules are manually created, limiting portability and flexibility [28], [32]. Updates in the future to accommodate changes in the language may have to be implemented manually, rather than in an automated manner.

The Naïve Bayes classifier demonstrated high performance. The main limitation observed with the use of this algorithm was its instability when groups with low frequency were included in the dataset. This behavior has been documented elsewhere [29]. The algorithm assumes that parameters are independent [32]. In this context the parameters were the binary occurrences, within each record, of the keywords from the dictionary built. Instability was however not observed to be due to occurrence of multiple keywords; rather it was associated with groups having small numbers of training examples. Due to the fact that the Naïve Bayes approach exhibits low transparency, it was not possible to track the specific mechanisms causing the problems observed in these low frequency categories, or to instigate measures to improve the way the algorithm was recording and using relationships between samples and the classification groups.

If transparency is not a limiting issue, that is, if domain knowledge experts are not required to understand and review the way by which the classifier is making decisions and classifying each instance, the Naïve Bayes algorithm can be an alternative to manually modified rules. Besides the high performance – though not as high as the custom set of rules – its implementation was the easiest of all algorithms evaluated, and automated updates can be planned by retraining the algorithm at regular intervals.

Nonmetric methods, such as Decision Trees, provide a “natural way to incorporate prior knowledge from human experts” [30]. However, this algorithm performed very poorly when small frequency groups were present; completely missing up to nine syndromic groups. Decision Trees were also very unstable to small changes on the data. This type of behaviour, in terms of training set sensitivity, has been well documented for Decision Trees [30].

**Figure 3:**
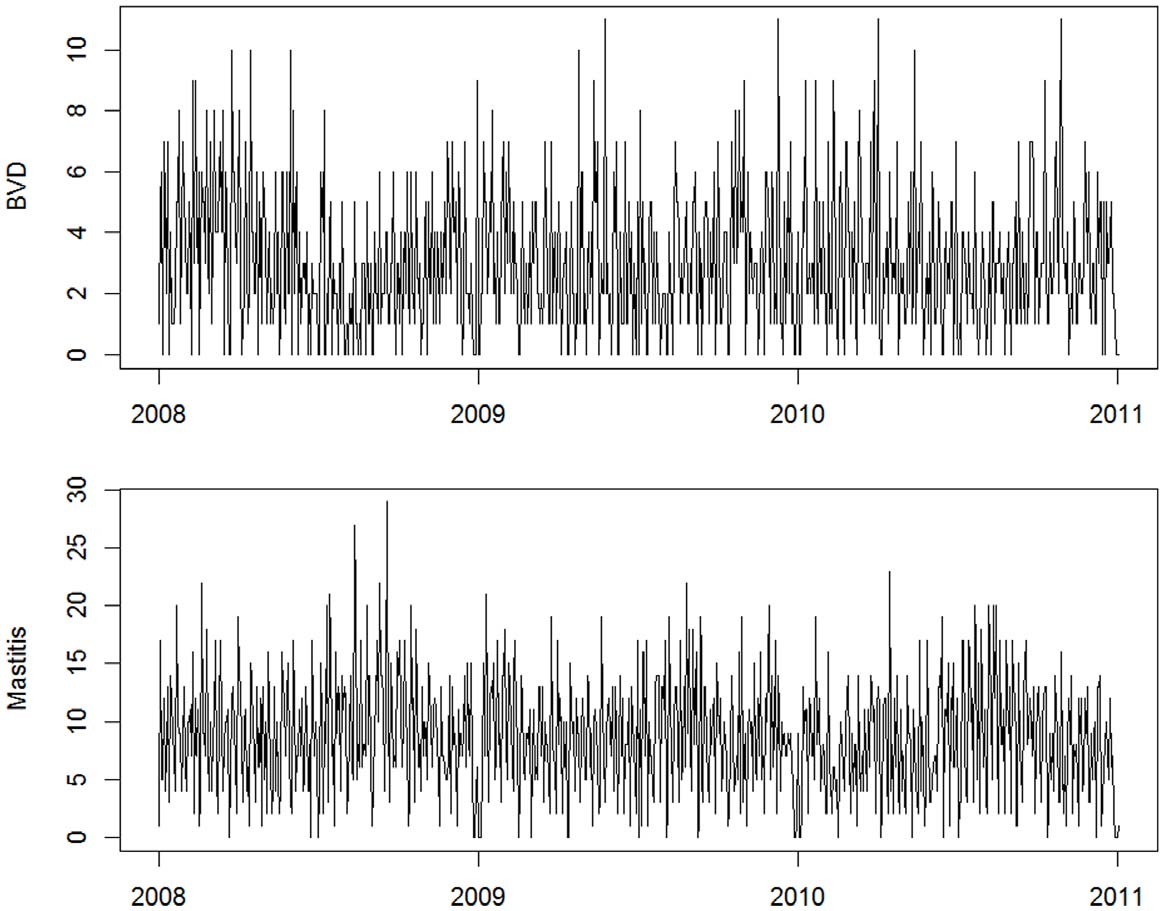
Daily counts of cases allocated to Bovine Viral Diarrhea (top) and Mastitis (bottom) syndromes.

The high performance reported in this study for the rule-based classifier refers to the algorithm’s ability to reproduce the manual classification of records provided by a human expert. This classification, however, is based on an active review of test orders and diagnostic specimens submitted. Clinical descriptions are not normally submitted by veterinarians, and were not available for use in the classification of records, which constitutes a limitation to the classification process. While the lack of clinical information is expected to reduce the precision and recall of the system in comparison to the actual syndromes observed by the veterinarians, the consistency of the classifier and its high accuracy in utilising the information that is available should allow the system to capture increases in the number of submissions across different syndromic groups. Figure 3 illustrates the time series of daily counts, constructed after data had been classified using the rule-based algorithm, for two syndromic groups with expected seasonal behaviour: Bovine Viral Diarrhea and Mastitis. The series reflect the expected seasonal patterns, which supports the conjecture that classified records successfully reflect real trends in the number of submissions for various syndromes.

The development of this system has been conducted at the request of the data providers and the Ontario Ministry of Agriculture Food and Rural Affairs, which is responsible for the animal surveillance activities in the province of Ontario. The system has benefited greatly from the automated extraction of surveillance information from this animal health database. As the information extraction was based on data already regularly submitted to the AHL without any requirement for passive or active collection of additional data, sustainability of the system is not expected to be an issue.

### Conclusion

Real-time monitoring of animal health data depends on establishing reliable models that reflect medical knowledge and that can be applied in an automated manner. Such models should be efficient, but also comprehensible to end users.

In this study the structured format of laboratory data, and the use of standard test codes, allowed for classification of approximately 75% of test requests into syndromic groups using direct mapping. For the remainder of the data, high accuracy (F_1_-macro = 0.997) was achieved through the use of a rule-based syndrome classifier. Induced rules were manually modified during the construction phase, but resulted in clear interpretability of decisions and resulting classification. While the use of rules was easy to implement and interpret, the construction of a dictionary of medically relevant terms and the manipulation of rules were time-consuming steps. Implementation of similar systems making use of other sources of laboratory data should be easier facilitated as standardized languages are more widely adopted in animal health laboratories, avoiding the repetition of this process for every new database.

The use of a custom rule set limits the potential for automatic revision of the classification model. Further research is required to establish internal validation rules, possibly based on the results available from historical data, in order to define automated ways to carry out model updates in the future.
